# A Double-Edged Sword: Extracellular Serine Proteases as Facilitators of Infection and Mediators of Immunity

**DOI:** 10.3390/molecules31040670

**Published:** 2026-02-15

**Authors:** Alua Shagirova, Maiya Allayarova, Aiya Makhanova, Amanbek Bekturgan, Timo Burster

**Affiliations:** Department of Biology, School of Sciences and Humanities, Nazarbayev University, Kabanbay Batyr Ave. 53, Astana 010000, Kazakhstan

**Keywords:** neutrophil serine proteases, cathepsin G, neutrophil elastase, furin, TMPRSS2

## Abstract

Serine proteases are a class of enzymes that orchestrate an immune response. These proteases can be hijacked by viruses to facilitate entry and spread, while simultaneously supporting the innate immune system in neutralizing pathogens. This review highlights the dual roles of exogenous serine proteases, emphasizing neutrophil serine proteases (NSPs) that facilitate viral entry and promote disease progression while also contributing to antiviral defense by degrading viral glycoproteins. Additionally, the potential to modulate serine protease activity to boost host defenses will be discussed, offering both significant challenges and new opportunities for therapeutic intervention.

## 1. Introduction

Serine proteases constitute a class of enzymes that serve as essential orchestrators of the immune response, maintaining a delicate balance within the host–pathogen interface. These proteases are characterized by a paradoxical duality: on the one hand, viruses subvert serine proteases to facilitate cellular entry and propagate infection, while on the other hand, they enable the innate immune system to mount effective defense mechanisms [1,2,3,4,5]. We will explore the complex and dichotomous capacity of exogenous serine proteases in host defense, with a particular focus on neutrophil-derived serine proteases. The discussion concludes with an analysis of the therapeutic potential of controlling serine protease activity, aiming to augment host defenses.

### 1.1. Biochemical Nature of Serine Proteases

Biochemically, serine proteases hydrolyze peptide bonds by employing a catalytic triad that generally consists of a serine, histidine, and aspartate residues. The serine amino acid in the catalytic center acts as a nucleophile, attacking the carbonyl carbon of the scissile peptide bond, while the histidine residue is responsible for the general base catalysis that facilitates the transfer of a proton from the catalytically active serine residue, and the aspartate moiety stabilizes the positively charged histidine [6]. Serine proteases typically exhibit a specific cleavage site, recognizing distinct amino acid sequences adjacent to the peptide bond, which is hydrolyzed. These catalytic enzymes are also categorized into families based on substrate specificity, such as trypsin-like (which cleaves at lysine or arginine residues) and chymotrypsin-like (which preferentially cleaves after aromatic amino acids) [6,7,8].

### 1.2. The Role of Neutrophils and Their Serine Proteases in Immunity

During acute inflammation, particularly in an acute inflammatory response, activated neutrophils release granules packed with serine proteases known as neutrophil serine proteases (NSPs) [9]. NSPs encompass neutrophil elastase (NE), cathepsin G (CatG), proteinase 3 (PR3), and neutrophil serine protease 4 (NSP4), which are tightly regulated by endogenous serine protease inhibitors, commonly known as serpins. Serpins play a crucial role in maintaining homeostasis in order to prevent excessive tissue damage and ensure a balanced inflammatory response [10,11,12]. Generally, serine proteases are integral in activating immune responses by processing precursor proteins; however, viruses can hijack serine proteases to maintain productive infection [3,5,13].

Once released, NSPs can transiently bind to the cell surface of a panel of different cells, enabling them to modulate an immune response in the local microenvironment [2,7]. For instance, CatG, NE, and to a lesser extent PR3 are basic proteins, positively charged amino acid residues, which allow them to bind electrostatically to the anionic sulfate groups of heparan sulfate and chondroitin sulfate proteoglycans, as shown for neutrophils [14]. Additionally, PR3 can utilize general charge-dependent electrostatic interactions with negatively charged proteoglycans; PR3 binds to the cell surface through a cluster of differentiation 177 (CD177) and phosphatidylserine [15,16,17]. When a virus approaches the cellular surface where NSPs are proteolytically active, the resulting interactions are complex and can be either deleterious to the host cell or protective, depending on the virus type, the timing of the interaction, the microenvironment, the specific protease involved, or the local protease concentration. For instance, low concentrations of NSPs can cleave host or viral surface molecules, thereby promoting viral attachment, fusion, internalization, and effectively increasing infectivity. In contrast, high protease activity or a pro-inflammatory environment can cause proteolytic degradation of viral particles, leading to shedding of viral receptors and activation of innate immune signaling pathways that have antiviral effects [2,3,10].

Recent studies have elucidated the critical involvement of cell surface serine proteases in viral pathogenesis and host antiviral defense mechanisms, as summarized in the following sections.

## 2. Proteases That Prime Viral Proteins to Facilitate Infection of the Host Cell

For many enveloped viruses, entry into a host cell requires a priming step where the proteolytic cleavage of a viral surface glycoprotein triggers membrane fusion. The localization of specific host proteases can significantly influence tissue tropism and viral spread. For example, some strains harbor a polybasic furin cleavage site within the fusion protein, which is cleaved by the ubiquitously expressed serine protease furin, allowing the virus to spread systemically and become more virulent [18].

Furin-mediated cleavage of the fusion protein has been observed across a wide range of enveloped virus families, including Orthomyxoviridae, such as the influenza A virus, which uses host furin to cleave viral glycoproteins, aiding entry into target cells by enabling fusion with the cell membrane [19]. Similarly, severe acute respiratory syndrome coronavirus 2 (SARS-CoV-2), which causes coronavirus disease 2019 (COVID-19), relies on host cell serine proteases, such as furin that cleaves the spike protein (S protein) at the polybasic S1/S2 boundary during viral assembly, and membrane-anchored serine proteases, including the cell surface transmembrane protease serine subtype 2 (TMPRSS2) which catalyzes the hydrolysis of the peptide bond at the S2′ site, generating the fusion peptide (FP), thereby facilitating cellular entry, and subsequent replication [3,20].

The next section will explore the specific molecular mechanisms that enable viral fusion and entry into host cells for selected viruses.

### 2.1. Influenza Virus

Priming the hemagglutinin (HA) protein through cleavage by host serine proteases is an essential step for influenza viral productive infection. Without this proteolytic cleavage, the HA protein stays in a non-functional precursor trimer (HA0), which cannot trigger the viral membrane fusion needed for the virus to enter a host cell. The influenza virus relies on host serine proteases to cleave the HA0 precursor into two subunits, HA1 and HA2, at the single cleavage site (CS). The cleavage separates the receptor-binding HA1 from the hydrophobic FP at the N-terminus of the HA2 subunit [21].

The membrane-anchored TMPRSS2, along with other proteases, is the main HA activator in human respiratory epithelium [22]. It has been demonstrated that genetic deletion of TMPRSS2 in mice provides resistance to lethal H1N1 and H7N9 influenza A strains, emphasizing its critical role in vivo [23]. Other types of TTSPs, such as TMPRSS4 and TMPRSS11D, also cleave HA, which depends on viral subtype and tissue distribution [24]. For seasonal influenza viruses and low-pathogenic avian influenza (LPAI) viruses, the HA0 protein is usually cleaved extracellularly by TMPRSS2 and TMPRSS11D. Controlled severity of influenza A virus disease and limited viral spread were seen in mice lacking TMPRSS2. Other TTSPs, such as TMPRSS4 and TMPRSS11D, can partially compensate in certain subtypes. Matriptase (ST14), another TTSP, also cleaves HA, alongside TMPRSS2 and TMPRSS4 [24]. On the other hand, highly pathogenic avian influenza (HPAI) viruses have an HA0 precursor with a polybasic cleavage site, which is cleaved by the ubiquitously expressed furin protease, allowing the viruses to infect a broader range of tissues and contribute to higher pathogenicity [25]. In conclusion, the LPAI virus, which has a monobasic HA0 cleavage site for cell surface TMPRSS2 and TMPRSS11D, is restricted to infecting respiratory and intestinal tissues, while HPAI viruses can spread to many organs due to the intracellular polybasic cleavage site for furin, causing severe, widespread disease.

### 2.2. HIV

The pro-viral effect of CatG, with a similar but less significant outcome by NE, promotes the multiplication of HIV-1 within macrophages. These immune cells exhibit increased susceptibility to acute HIV infection following pretreatment with CatG, whereas CD4^+^ T cells remain unaffected. It was suggested that CatG interacts with cell surface receptors or activates specific signaling pathways that prime macrophages for more efficient viral infection. The mechanism relies on Gi-protein-dependent signaling mechanisms. Furthermore, CatG functions as a chemoattractant, recruiting monocytes and macrophages to sites of inflammation, thereby augmenting the pool of potential target cells for HIV. In summary, while CatG engages with host cellular factors, CatG does not directly interact with the viral particles [26]. Notably, furin controls viral activation by directly maturing the HIV-1 envelope glycoprotein 160 (gp160). Unlike the cell-surface triggers needed by influenza, this proteolytic cleavage into the receptor-binding (gp120) and transmembrane (gp41) subunits happens inside the producer cell within the secretory pathway [27] and renders the virus independent from further proteolytic activation upon entry into a new host cell.

### 2.3. The Hepatitis C Virus (HCV)

HCV infection of hepatocytes is a tightly regulated process that depends on coordinated interactions with attachment factors and essential entry factors, such as CD81, scavenger receptor class B type I, claudin-1, and occludin, followed by clathrin-mediated endocytosis [28]. Unlike influenza HA or coronavirus S protein, HCV entry is primed by receptor engagement at the cell surface for subsequent low-pH-dependent fusion in endosomes, usually without a single required protease cleavage step at the cell membrane [29,30]. However, HCV can use host cell surface serine proteases to assist viral entry and modulate the immune response. Among these, the activity of a trypsin-like serine protease (TMPRSS2 in this case) activates HCV infection at the post-binding and entry stages through proteolytic activation of the HCV envelope glycoprotein E2, which promotes fusion and entry into hepatocytes [31]. Additionally, HCV infection can modify the protease-protease inhibitor balance in infected cells. Notably, hepatitis B and hepatitis C virus replication increases the expression of serine protease inhibitor Kazal-type 1, leading to cellular resistance to serine protease-dependent apoptosis [32]. While the general HCV invasion process is mainly driven by host cell pathways, the activity of cell surface serine proteases can still impact infection efficiency at the initial stage, influencing viral spread and disease progression [31,32].

### 2.4. SARS-CoV-2 S Protein Facilitates Entry into the Host Cell

SARS-CoV-2 relies on host cell surface receptor angiotensin-converting enzyme 2 (ACE2) and serine proteases for entry into target cells, which is mediated by the S protein. The S protein consists of two functional sites, the S1 for binding to ACE2 and the S2′ for fusion of the viral envelope with the host cell membrane. The S1/S2 interface at _685_RS_686_ (furin cleavage motive is RRAR/S) is hydrolyzed by the serine protease furin, and the resulting S1 subunit is non-covalently associated with the remaining S2 part, which was generated in the infected producer cells before the virions were released [33,34,35]. In contrast, the S2′ part at _815_RS_816_ is cleaved by TMPRSS2, generating the hydrophobic FP that mediates SARS-CoV-2 fusion with the host cell membrane [36]. Beyond the canonical S protein priming by furin and TMPRSS2, several other host proteases have been reported to be co-opted, depending on the tissue and viral variant, including TMPRSS1, TMPRSS4, TMPRSS11D, TMPRSS13, and matriptase [37,38,39,40,41]. In an alternative entry mechanism, SARS-CoV-2 can access the target cell through a TMPRSS2-independent endocytosis pathway, where the cysteine protease cathepsin L (CatL) catalyzes the cleavage of the S2′ site, thereby facilitating the fusion of SARS-CoV-2 with the endosomal membrane and the subsequent release of its genetic material into the cytosol [3,42,43].

Besides TMPRSS2 and furin, which regulate coronavirus entry into host cells, NE has also been shown to modulate viral entry and the immune response at the airway surface. Originally, it was identified that porcine elastase can activate the SARS-CoV-1 S protein, playing a critical role in fusion activation [44,45], and a recent study by Yamamoto et al. demonstrated that porcine elastase is capable of cleaving the S protein (SARS-CoV-2) of bat-derived sarbecoviruses RaTG13 and Khosta-2, promoting syncytium formation and enhancing TMPRSS-2-dependent entry into human cells [46]. These findings demonstrate that neutrophil-rich inflamed airways and NE deposition at the epithelial surface can create a proteolytic microenvironment that favors S protein activation for viral fusion with the host cell membrane.

### 2.5. Furin Also Cleaves at S2′ and Generates the Fusion Peptide

**Figure 1:**
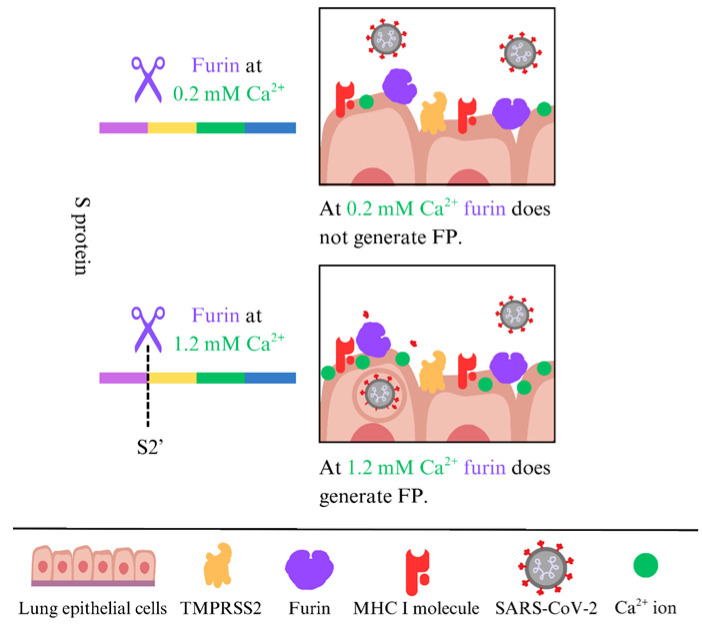
Cleavage of the S2′ site by furin depends on the Ca^2+^ concentration.

The peptide bond at the S1/S2 interface _685_RS_686_ is hydrolyzed by furin [47]. Recently, we demonstrated that furin, with a final concentration of 0.2 mM Ca^2+^, cleaves at the S1/S2 interface at _685_RS_686_. However, the S2′ site at _815_RS_816_, which generates the hydrophobic FP, is not cleaved by furin at 0.2 mM Ca^2+^; higher concentrations are needed (1.2 mM) [48] (Figure 1). Generally, the level of free Ca^2+^ in the extracellular fluid is about 1 mM, compared to the resting intracellular level of roughly 100 nM [49]. These data indicate that only the S1/S2 interface at _685_RS_686_ is hydrolyzed by furin, and the resulting S1 subunit remains non-covalently attached to the remaining S2 part, which is generated in the infected producer cells before the virions are released [33,34,35]. From the trans-Golgi network, where furin resides, furin can traffic through endosomal compartments to the cell surface and is tethered by actin-binding protein-280 [50,51]. At the cell surface, TMPRSS2 is the primary protease that generates the fusion peptide, but furin can also cleave at the S2′ site, as can TMPRSS2 [52].

## 3. Innate Immunity Involves Proteases Targeting and Neutralizing the Virus, Thereby Safeguarding the Host Cell

Conversely, the innate immune system, as the first line of defense, has developed strategies to counter pathogen invasion. For example, neutrophils secrete serine proteases that proteolytically degrade viral glycoproteins, neutralizing pathogens and preventing them from spreading within the body [53]. The principal defensive function of cell surface NSPs against viral pathogens is the capacity to facilitate the proteolytic degradation of viral constituents, thus providing protection against infection [54]. The mechanism will be further elucidated in the following section.

### 3.1. Influenza Virus

During influenza infection, neutrophils are rapidly recruited to the infected airway and contribute to antiviral defense through degranulation and the formation of neutrophil extracellular traps (NETs). NETs are reticular extracellular structures made of DNA, histones, and antimicrobial proteins, including NE, CatG, and myeloperoxidase (MPO), and they are released in response to viral infection [55]. NE contributes to antiviral defense primarily by driving NET formation. When activated, NE is released from azurophilic granules and translocates to the nucleus, where it cleaves histones and causes chromatin decondensation, with MPO assisting in this process [56]. Following NET release, NE and MPO are externalized within the NET scaffold at the epithelial surface, creating a localized protease-rich microenvironment that traps viral particles and can concentrate antimicrobial factors, thereby limiting local spread and subsequent entry into neighboring target cells [55]. However, a protease-rich NET response can become harmful when excessive or sustained. In influenza pneumonitis models, marked neutrophil influx and NET accumulation colocalize with tissue injury and associate with the disruption of the alveolar-capillary barrier, indicating that NET-associated enzymes and oxidant systems can aggravate lung pathology during dysregulated inflammation [57]. Thus, localized proteolysis may restrict viral spread early on, but prolonged or excessive activity can cause tissue damage and worsen disease severity beyond direct viral cytopathicity [57].

### 3.2. HIV

The V3 loop of HIV gp120 inhibits membrane-associated CatG on U-937 cells [58,59], and CatG has been shown to modulate HIV infection by processing the co-receptor for HIV RANTES/CCL5 at the N-terminus, generating a truncated 4-68 variant with decreased chemotactic activity and the ability to block viral binding, thereby preventing HIV from entering host cells [60], serving as an immune evasion mechanism. Prolonged or continuous exposure to CatG subsequent to the initial infection suppresses HIV replication within macrophages, indicating that CatG can function as an inhibitor of HIV infection. However, as discussed in Section 2.2, the role of CatG seems to be contingent upon specific contextual factors, notably the precise timing and duration of exposure. In fact, extended exposure to CatG underscores the critical significance of regulating CatG’s impact on HIV replication [26].

### 3.3. Proteases That Degrade the S Protein of SARS-CoV-2 to Block Cell Entry

Exogenous expression of TMPRSS11D enhances the SARS-CoV-2 life cycle [38], whereas soluble mature TMPRSS11D in the extracellular environment is linked to antiviral activity; however, the emergence of new variants (Delta and Omicron) has shown resistance to the anti-SARS-CoV-2 capacity of TMPRSS11D [61]. TMPRSS11D can serve as both a key factor in viral spread and an innate defense molecule, depending on its form, location, and the specific viral strain.

**Figure 2:**
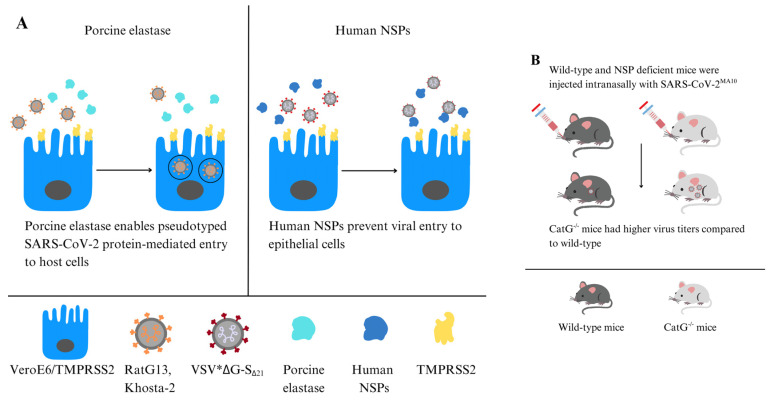
NE can enhance or inhibit SARS-CoV-2 infection. (**A**) Porcine elastase facilitates host cell entry mediated by pseudo-typed SARS-CoV-2 S protein (**left** panel) [46]. NSPs digest the SARS-CoV-2 S protein, while CatG prevents the virus from entering host cells in Vero cells expressing TMPRSS2 (**right** panel). (**B**) NE-/- and PR3-/- mice experienced significant weight loss compared to WT mice after CoV-2^MA10^ infection. CatG-/- mice showed increased viral titers in the lungs in contrast to controls [54].

In the innate immune system, the mechanism by which serine proteases degrade the FP underscores their potential role in antiviral defense. NE and CatG can directly interact with the viral glycoprotein by cleaving the S protein, effectively rendering the virus inactive, as enzymatic activity disrupts the viral fusion process. Indeed, surface proteases assist in defending against viral infections, as the variability in SARS-CoV-2 infection outcomes between individuals may be attributed to differences in proteases responsible for viral degradation [62]. Benarafa and colleagues showed that NSPs degrade the SARS-CoV-2 S protein, reducing its stability, and inhibit viral entry into host cells, such as Vero cells expressing TMPRSS2 (Figure 2A). The absence of NE and PR3 directly affected the signs associated with SARS-CoV-2^MA10^ infection, as NE-/- and PR3-/- mice experienced significantly greater weight loss compared to WT mice after infection. CatG-/- mice had notably higher viral titers in the lungs than WT mice. Double knockout mice, NE-/- and CatG-/-, were the most severely affected, showing the greatest weight loss, increased inflammation, and lung tissue damage, indicating antiviral and anti-inflammatory roles of NSPs during SARS-CoV-2 infection. NE can also cleave other cell surface proteins, such as the ACE2 receptor used by SARS-CoV-2 to enter the host cell. This cleavage reduces the availability of receptors for viral binding, further hindering viral entry. In vivo studies showed that NE knockout (-/-) and CatG-/- mice increased pulmonary inflammation and more severe lung pathology than wild-type controls [54] (Figure 2B).

**Figure 3:**
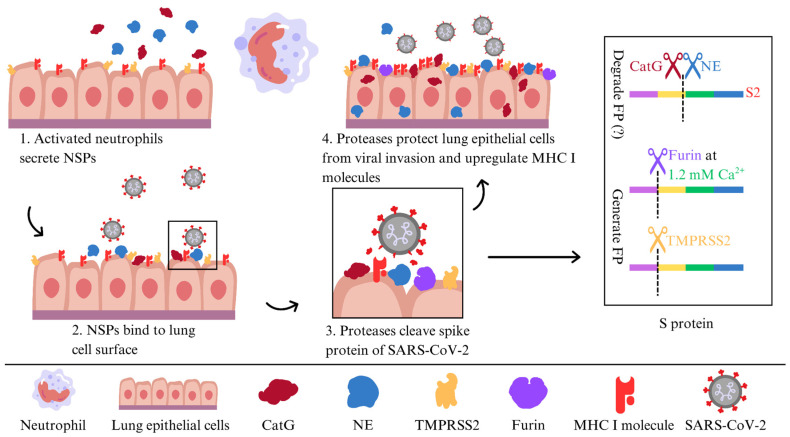
A model of NE and CatG binding to the surface of lung epithelial cells to defend against SARS-CoV-2 infection.

Furthermore, we revealed the pivotal role of NE and CatG in fortifying target cells, possibly against viral entry, through their binding capacity and being proteolytically active on the surface of the human alveolar basal epithelial cell line (A549) and the human lung epithelial-like cell line (H1299). Additionally, NE and CatG increase cell-surface levels of major histocompatibility complex class I (MHC I) molecules, which are crucial for presenting the endogenous antigenic repertoire to T cells. Moreover, our findings indicate that NE and CatG degrade the FP of SARS-CoV-2, as demonstrated by using a peptide spanning the S2′ site [48] (Figure 3). The augmentation of MHC I surface expression potentially arises from distinct mechanisms: NE is internalized via clathrin-mediated endocytosis [63] and might slow down MHC I turnover, leading to increased cell surface MHC I molecules [64,65], whereas CatG probably interacts with the protease-activated receptor 1 (PAR1) to promote recycling of MHC I back to the cell surface [66]. Another possibility is that serine proteases can modulate host cell receptors that viruses use for entry. By cleaving these receptors, serine proteases can hinder viral binding and decrease susceptibility to infection. Some serine proteases also regulate apoptotic pathways in response to viral infections. By regulating the apoptotic process, these enzymes can affect the viability of infected cells and constrain viral dissemination [53].

### 3.4. NSPs Can Either Promote or Prevent Infection by SARS-CoV-2

Contradictory evidence has been reported regarding NE. On one hand, porcine elastase facilitates host cell entry mediated by pseudo-typed SARS-CoV-2 S protein and exhibits proviral effects [46]. Interestingly, porcine elastase also supports TMPRSS2 priming of bat RaTG13 and Khosta-2 (pseudo-typed bat SARS-like CoV) for viral fusion with the membrane, especially since low TMPRSS2 expression does not show this effect. Consequently, porcine elastase and TMPRSS2 synergistically facilitate cell entry mediated by the S proteins of bat RaTG13 and Khosta-2 [46] (Figure 2A). Moreover, neutrophil–epithelial interactions induce a pro-inflammatory state, enhancing SARS-CoV-2 infectivity by compromising epithelial barriers and exposing basal cells. Neutrophils can indirectly promote viral spread by weakening tissue defenses [67].

On the other hand, NE and CatG are defenders against viral infection as discussed in Section 3.3 [54]. Additionally, it has been shown that NSPs modulate the initial attachment phase of SARS-CoV-2 by targeting the viral receptor rather than directly interacting with the S protein, due to NE cleavage of the ACE2 ectodomain on human bronchial epithelial cells, thereby decreasing S protein binding affinity. Consequently, this reduction hampers viral attachment and entry [68]. This indicates a protective mechanism in which increased levels of NE in airway secretions trigger receptor shedding, thereby reducing viral infectivity.

NE and CatG have multiple cleavage sites within the S protein [54,69,70]. Besides the importance of the cleavage site itself, the three-dimensional structure of the S protein trimer (accessible by proteases), the timing of protease action, the source of proteases (which should be of human origin when using human host cells), species of host cells, the precise cleavage site _815_RS_816_ to generate the FP (which is not the case for NE or CatG), and the specific SARS-CoV strain used are also significant. Even though the virus undergoes mutations that result in amino acid substitutions, eliminating all cleavage sites that could reduce the destructive capacity of NE or CatG might be unlikely. Thus, it is highly probable that NE and CatG are responsible for degrading the SARS-CoV-2 S protein, thereby impeding viral entry into host cells. This is consistent with the innate immune response, wherein neutrophils release NSPs and can bind to the surface of host cells, serving as a protective mechanism against SARS-CoV-2 infection.

During an innate immune response, administering proteases as prospective therapeutic agents or components that boost the catalytic efficiency of NSPs, provoking a transiently elevated proteolytic activity of NE and CatG, may be advantageous in neutralizing the virus; however, excessive enzymatic activity can precipitate pathological conditions in severe cases. For example, high levels of CatG are associated with detrimental inflammation and thrombogenesis, which are pivotal features of severe COVID-19 complications [71,72].

### 3.5. T Cells Are Armed with CatG, Possibly to Defend Themselves Against Viruses at the Site of Infection

CatG is present on the surface of immune cells, including T cells [73,74]. CatG bound to the surface of T cells may act as a protective barrier, potentially reducing the cells’ susceptibility to viral infections. Moreover, NE and CatG significantly increase the expression of MHC I molecules on a CD4^+^ T cell line (Jurkat cells), suggesting possible differences in how antigenic peptides are presented compared to other cell types, and imply that immune cells in transit may need enhanced surveillance to prevent viral spread [48]. Nevertheless, studies have shown that SARS-CoV-2 can directly infect human CD4^+^ T helper cells and cause their depletion in COVID-19, but not CD8^+^ T cells, indicating that viral entry is mediated by the CD4 molecule [75]. It is noteworthy that T cells do not express the cell surface protease TMPRSS2 [76], but T cells do express furin [18,77], resulting in the generation of the FP. One plausible explanation for infection is the imbalance of serpin concentrations in the microenvironment, which might inhibit cell-bound NSPs on T cells. Levels of serpins during inflammation and their inhibitory capacity will also be discussed in the subsequent section.

## 4. Long COVID, Potentially Regulated by NSPs and Serpins

Long COVID refers to the lingering symptoms experienced by individuals after their initial recovery from COVID-19, which has raised concerns about the long-term impact of SARS-CoV-2. Long COVID is characterized by symptoms that can persist for weeks or months after the initial infection. These symptoms include conditions such as cardiovascular, thrombotic, and cerebrovascular disease, as well as myalgic encephalomyelitis/chronic fatigue syndrome and postural orthostatic tachycardia syndrome [78,79]. The severity and duration of these symptoms can vary greatly between individuals, making it a complex and challenging condition to manage in daily life. The pathogenesis of Long COVID has been linked to hypotheses including persistent SARS-CoV-2 in tissues, reactivation of underlying pathogens (Epstein–Barr virus and human herpesvirus 6), autoimmunity, and immune dysregulation [79]. Notably, pharmacological inhibition of NE with sivelestat significantly reduces chronic lung inflammation [80], and NE, S100B, and related inflammatory markers are strongly linked to long-term respiratory dysfunction in patients months after SARS-CoV-2 infection [81]. Elevated levels of CatG and uncontrolled release of CatG by neutrophils contribute to detrimental inflammation and thrombogenesis, which are characteristic outcomes of severe COVID-19 [71,72].

A critical aspect of the immune response to SARS-CoV-2 involves protease-mediated protein degradation, with neutrophils initiating a cellular immune response against pathogens. Of particular interest is the role of NE and CatG, which can bind to the cell surface and exhibit proteolytic activity to fortify target cells [48] against viral invasion [54]. The protective function of the serine protease inhibitor SerpinA1 (also called alpha1-antitrypsin) in COVID-19 has been discussed, although this remains a topic of controversy [82,83]. In general, SerpinA1 is known to inhibit CatG, NE, PR3, and TMPRSS2, and is typically upregulated in response to managing inflammation and tissue damage, which is key after the immune system returns to homeostasis. Given the controversy surrounding the role of SerpinA1 in COVID-19, it is important to delve deeper into the regulation of serpin protease inhibitors, especially SerpinA3, which is a more potent CatG inhibitor and has been found to be upregulated in critical COVID-19 patients [84,85]. The dysregulation of these protease inhibitors, particularly during viral infections, raises intriguing questions about their potential impact on disease severity. One scenario to examine more closely is a potential decrease in SerpinA3 levels during viral infections, particularly in asymptomatic individuals, which could lead to reduced inhibition of CatG to enhance CatG-mediated viral clearance [48].

Besides proteolytic functions, genetic polymorphisms in serine protease genes influence host susceptibility to severe viral infections. A cross-sectional investigation involving over 1500 COVID-19 patients identified several single-nucleotide polymorphisms (SNPs) within the TMPRSS2 and SERPINE1 genes. The TMPRSS2 rs75603675 AA genotype has been correlated with increased mortality risk, whereas specific SERPINE1 variants, such as rs2227667, appeared to exert a protective effect. In contrast, the SERPINE1 rs2227692 T allele and TT genotype increased the risk of mortality and elevated D-dimer levels, implicating a role in hypercoagulability. These findings highlight that host genetic architecture influences viral entry mechanisms mediated by surface protease activity and subsequent inflammatory and thrombotic pathways. A more nuanced understanding of such genetic determinants could guide the development of therapies targeting serine protease pathways [86].

## 5. Application of Serine Protease Inhibitors or Activators

Understanding serine proteases on the cell surface has revealed new possibilities for treating viral infections. For example, inhibiting TMPRSS2, due to its role in facilitating the entry of several viruses into cells, could be a strategy to reduce infection rates by developing specific inhibitors for TMPRSS2 [22]. Therefore, selective TMPRSS2 inhibitors or the application of a broad-spectrum of protease inhibitors targeting the airway epithelium, where viral replication occurs, are implicated for drug development [87]. TMPRSS2 possesses the capacity to cleave the SARS-CoV-2 S protein at the S2′ site, a critical step that unveils the FP and facilitates membrane fusion at the cellular surface; however, inhibition of TMPRSS2 redirects the virus to alternative entry pathways, such as the cathepsin-mediated endosomal route [34]. Inhibition of TMPRSS2 by camostat or the more effective inhibitor nafamostat [88] was examined in a meta-analysis of randomized clinical trials. Camostat and nafamostat are well tolerated, although efficacy signals were inconsistent across different study populations. Some subgroup analyses favored nafamostat, but no definitive clinical benefit was established [89]. Randomized controlled trials of camostat, covering mild-to-severe COVID-19 cases, confirmed safety but did not demonstrate consistent therapeutic benefits [90], raising questions about the timing of the intervention limiting the effectiveness of camostat [91].

Aprotinin is a broad-spectrum inhibitor of serine proteases, some of which are involved in SARS-CoV-2 S protein activation and in the regulation of host cell-surface proteins. The ATAC Phase III Randomized Controlled Trial evaluated the effectiveness of inhaled aprotinin in patients with COVID-19 pneumonia [92]. Patients who received aprotinin in addition to the standard care experienced significant improvement. Aprotinin recipients had a 5-day reduction in hospital stay, a two-day shorter treatment duration, and were discharged earlier, with a hazard ratio for discharge of 2.19 (95% CI: 1.182–4.047; *p* = 0.013). In addition, the group showed reduced oxygen requirements and reported no significant adverse events [92]. A1-Antitrypsin (A1AT), an endogenous serpin that inhibits NSPs, is also known to be a direct inhibitor of TMPRSS2, which can prevent SARS-CoV-2 entry in airway epithelial models [93]. NE and CatG degrade the SARS-CoV-2 S protein, deactivate the virus, and reduce lung inflammation; broad protease inhibition by A1AT can negate NSP-mediated antiviral effect [54]. A1AT can reduce tissue damage during infection and also restrict the entry of viruses into cells by inhibiting TMPRSS2. However, inhibiting NSPs, which are part of the body’s antiviral defenses, risks removing an early antiviral barrier crucial in the initial stages of infection. This delicate balance emphasizes the importance of precise timing when considering the therapeutic use of aprotinin, A1AT, or NSP inhibitors to maximize benefits and reduce potential drawbacks.

Strikingly, eosinophil cationic protein (ECP) augments the proteolytic function of NE [94] or, as we demonstrated, lactoferrin (LF) enhances the proteolytic activity of CatG [95]. Furthermore, LF and its derivative peptides, such as lactoferricin, have direct antiviral effects by inhibiting TMPRSS2 activity, blocking S protein priming, and reducing SARS-CoV-2 infection in susceptible cell lines [96]. Moreover, a biophysical study demonstrated that full-length human LF limits viral infection by masking the host receptor, binding to ACE2 instead of the spike receptor-binding domain (RBD). Docking revealed three contact sites on ACE2, indicating multiple ACE2-centered mechanisms of entry inhibition [97]. Alves et al. showed that both apo- and holo-bovine LF exhibit nearly a 70% reduction in viral entry across ancestral and Omicron variants in vitro [98]. Interestingly, bovine LF was also shown to directly bind the RBD of the S protein and inhibit the viral RNA-dependent RNA polymerase (RdRp) in vitro, reducing lung viral loads in hamsters [99]. A clinical trial involving hospitalized patients with moderate-to-severe COVID-19 found LF to be safe and well tolerated; however, the trial did not show any significant improvement in clinical outcomes or inflammatory markers, suggesting that efficacy may depend on the disease stage, timing (patients took LF later in the disease, approximately 6 days after symptom onset), and means of LF delivery [100]. These findings suggest that LF and related peptides are potent natural adjuncts that can reduce SARS-CoV-2 infection and help decrease excessive inflammation but surprisingly did not lead to clinical improvement.

An initial enhancement of the proteolytic activity of NSPs combined with inhibition of TMPRSS2 and furin may offer certain advantages; however, prolonged proteolytic activity poses the potential for adverse effects.

## 6. Conclusions

Host cell-surface serine proteases have complex, often dual, roles in viral infections. Viruses can hijack these proteases to assist entry and spread, or they can be deployed by the innate immune system to neutralize pathogens. Consequently, this intricate interaction reflects the evolutionary arms race between the host and the virus, with the potential to modulate serine protease activity, initially boosting host defenses and offering insights into innovative antiviral strategies. To counteract immune evasion, components that enhance the catalytic efficiency of NSPs are indicated. For instance, ECP increases the proteolytic activity of NE [94], and LF for CatG [95]. On the other hand, the potential for off-target effects when targeting serine proteases therapeutically requires careful study. Depending on the individual’s medical condition or infection status, exploring the potential of combination therapies that include serine protease inhibitors and/or activators alongside traditional antiviral drugs may improve treatment efficacy. Finally, further research is needed to fully understand the dual roles of serine proteases in immunity and the potential functions that proteases may have in preventing viral infections. Exploring how serine protease activity can be modulated may lead to innovative therapeutic approaches in immunotherapy.

## Data Availability

No new data were created or analyzed in this study.

## Abbreviations

The following abbreviations are used in this manuscript:

NSPs	Neutrophil serine proteases
CatG	Cathepsin G
CS	Cleavage site
NE	Neutrophil elastase
PR3	Proteinase 3
NSP4	Neutrophil serine protease 4
CD177	Cluster of differentiation 177
SARS-CoV-2	Severe acute respiratory syndrome coronavirus 2
COVID-19	Coronavirus disease 2019
S protein	Spike protein
TMPRSS2	Transmembrane protease serine 2
FP	Fusion peptide
HA	Hemagglutinin
TTSPs	Type II transmembrane serine proteases
TMPRSS4	Transmembrane protease serine 4
TMPRSS11D	Transmembrane protease serine 11D
LPAI	Low-pathogenic avian influenza
ST14	Suppressor of tumorigenicity 14 protein (matriptase)
HPAI	Highly pathogenic avian influenza
HIV-1	Human immunodeficiency virus type 1
CD4^+^	CD4 positive T helper cell
RANTES	Regulated on activation, normal T cell expressed and secreted
CCL5	Chemokine (C-C motif) ligand 5
HCV	Hepatitis C virus
ACE2	Angiotensin-converting enzyme 2
CatL	Cathepsin L
MOP	Myeloperoxidase
MHC I	Major histocompatibility complex class I
NET	Neutrophil extracellular trap
PAR1	Protease-activated receptor 1
CD8^+^	CD8 positive T cell
SerpinA1	Serine protease inhibitor A1
SerpinA3	Serine protease inhibitor A3
SNPs	Single-nucleotide polymorphisms
A1AT	A1-Antitrypsin
ECP	Eosinophil cationic protein
LF	Lactoferrin
RBD	Receptor-binding domain
RdRp	RNA-dependent RNA polymerase
